# Current News

**Published:** 1901-09

**Authors:** 


### Current News.

### TEXAS STATE DENTAL ASSOCIATION.

The Texas State Dental Association met at Sherman, May 14, 15, and 16, 1901, with a good attendance, and a splendid meeting resulted. Waco was selected as the place for the next meeting, 1902. The following officers were elected:

President, Dr. H. L. Pearson, McKinney; First Vice-President, Dr. J. G. Fife, Dallas; Second Vice-President, Dr. Thomas P. Williams, Houston; Secretary and Treasurer, Dr. Bush Jones, Dallas; Curator of Museum, Dr. A. F. Sontag, Waco.

*Executive Committee.*—Dr. Sam G. Duff, Chairman, Greenville; Dr. E. F. Comegys, Gainesville; Dr. W. R. Rathbone, Cuero.

BUSH JONES,
*Secretary.*

---

### CENTRAL DENTAL ASSOCIATION OF NORTHERN NEW JERSEY.

AT the annual meeting of the Central Dental Association of Northern New Jersey, held on the evening of February 18, 1901, the election resulted as follows: President, Frank G. Gregory, Newark; Vice-President, J. W. Fisher, East Orange; Secretary, Frederick W. Stevens, Newark; Treasurer, Charles A. Meeker, Newark.

FREDERICK W. STEVENS,
*Secretary.*

---

### ILLINOIS STATE DENTAL SOCIETY.

FOLLOWING is a list of officers and committees elected for the ensuing year at the last annual meeting of the Illinois State Dental Society:

President, M. L. Hanaford, Rockford; Vice-President, J. E. Hinkins, Chicago; Secretary, A. H. Peck, 92 State Street, Chicago; Treasurer, C. N. Johnson, Chicago; Librarian, J. T. Cummins, Metropolis City.

*Executive Council.*—J. R. Rayburn, Fairbury; W. E. Holland, Jerseyville; J. G. Reid, Chicago.

*Executive Committee.*—J. W. Cormany, Mt. Carroll.

*Publication Committee.*—A. H. Peck, C. N. Johnson, Edmond Noyes, all of Chicago.

*Board of Examiners.*—Edmond Noyes, Chicago, to succeed C. M. Robbins, term expired.

*Committee on Dental Science and Literature.*—G. V. Black, Chicago.

*Committee on Dental Art and Invention.*—H. J. Goslee, Chicago.

*Committee on Infraction of Code of Ethics.*—T. L. Gilmer, Chicago; A. S. Waltz, Decatur; O. L. Frazee, Springfield.

*Supervisor of Clinics.*—D. M. Gallie, Chicago.

*Committee on Local Arrangements.*—O. L. Frazee, Chairman; E. F. Hazell, T. P. Donelan, Assistants, all of Springfield.

A. H. Peck,
*Secretary.*

---

### ANNUAL MEETING OF THE MISSISSIPPI VALLEY MEDICAL ASSOCIATION.

The next annual meeting of the Mississippi Valley Medical Association, under the presidency of Dr. A. H. Cordier, of Kansas City, bids fair to eclipse all previous ones in attendance as well as scientific merit, as the following preliminary programme will show.

Unusual railroad rates have been obtained for this meeting: a one-fare rate by way of Cleveland, which will enable those taking advantage of these rates to obtain an extension of tickets to October 8 for attendance upon the Buffalo Exposition; a one-and-a-third-fare rate on the certificate plan will be in effect *via* Detroit, Sandusky, and Toledo, with extension of return limit for only three days after the meeting.

Put-in-Bay is an ideal place of meeting, the Hotel Victory a magnificent meeting site.

The address in Medicine will be made by Dr. Frank Billings, of Chicago; the address in Surgery by Dr. Reginald Sayre, of New York City. The Association is to be congratulated on the selection of these two orators, who will acquit themselves in a most scholarly manner.

The annual banquet will be held on the evening of the first day, September 12; on the second, the evening will be given up to the reading of several papers with stereopticon exhibits and demonstrations, the President's address and the annual orations being delivered on the three mornings of the meeting.

The profession is cordially invited to attend this meeting.

No title can be received after August 20 for publication on the final programme.

